# Resveratrol reverses multidrug resistance in human breast cancer doxorubicin-resistant cells

**DOI:** 10.3892/etm.2014.1662

**Published:** 2014-04-03

**Authors:** FANG HUANG, XIAO-NAN WU, JIE CHEN, WEN-XIANG WANG, ZU-FU LU

**Affiliations:** 1 Department of Nutrition and Health Care, School of Public Health, Fujian Medical University, Fuzhou, Fujian 350004, P.R. China; 2 School of AMME, The University of Sydney, Darlington, New South Wales 2050, Australia

**Keywords:** resveratrol, multidrug resistance, human breast cancer doxorubicin-resistant cell

## Abstract

Although its mechanisms remain unidentified, resveratrol (trans-3,4′,5-trihydroxystilbene; RES), which is an active, low molecular-weight compound, possesses a unique antitumor function and is capable of enhancing the cytotoxicity of doxorubicin (DOX) within solid tumor cells. RES is hypothesized to exert these effects by reversing the multidrug resistance (MDR) of the cancer cells in response to chemotherapeutic agents. The aim of the present study was to investigate the reversal effect of RES on MDR in human breast cancer DOX-resistant (MCF-7/DOX) cells and investigate the underlying mechanisms of RES. The results demonstrated that RES inhibited the proliferation of MCF-7/DOX and MCF-7 cells in a dose-dependent manner. Moreover, RES enhanced the cytotoxicity of DOX on MCF-7/DOX cells and the reversal index of RES treatment was demonstrated to be significantly higher when compared with that of the group without RES treatment. In addition, RES was observed to reverse the MDR of the MCF-7/DOX cells and elevate the concentration of DOX in the MCF-7/DOX cells. Furthermore, RES was identified to significantly downregulate the MDR-1 gene and P-glycoprotein expression levels. Reversing MDR, via the downregulation of MDR-1 expression, was concluded to be a mechanism of RES, which enables the unique antitumor function of this polypeptide. Therefore, the present study indicated that RES may be a novel MDR reversal agent for the treatment of breast cancer.

## Introduction

Breast cancer is a frequently diagnosed type of cancer and is a predominant cause of mortality among females worldwide (1). Multidrug resistance (MDR) and chemotherapeutic agent toxicity are two predominant obstacles to the success of chemotherapy (2–4). The molecular mechanisms that lead to MDR include, the activation of transport and detoxification systems, enhancement of target repair activities, alteration of drug targets and dysregulation of cell death pathways (5,6). MDR can result from the overexpression of transporter proteins, such as P-glycoprotein (P-gP) and other breast cancer resistance proteins. P-gP is a 170 kDa plasma membrane protein that facilitates the efflux of chemotherapeutic agents from tumor cells. P-gP is coded by the MDR-1 gene and functions as an energy-dependent efflux pump, which rapidly extrudes a variety of anticancer drugs from target cancer cells, thus reducing drug cytotoxicity (7–9). A number of drugs have been reported to overcome MDR effectively and are, therefore, considered for use with P-gP inhibitors in conjunction with other anticancer agents during tumor treatment (10,11). However, the side effects of these agents compromise their clinical application. Thus, the identification of novel agents with low toxicity is necessary to satisfy the requirement in clinical applications.

Resveratrol (trans-3,4′,5-trihydroxystilbene; RES), a compound obtained primarily from root extracts of the oriental plant, *Polygonum cuspidatum* and from red grapes, has been identified by previous studies as possessing a strong chemopreventive effect against the development of breast cancer (12–15). Our previous studies demonstrated that RES, quercetin or ferulic acid alone are able to inhibit human breast cancer doxorubicin (DOX)-resistant (MCF-7/DOX) cell proliferation; moreover, RES more efficiently inhibited cancer cell proliferation than quercetin or fumaric acid (16). Although RES was reportedly capable of enhancing the cytotoxicity of anticancer agents by increasing the intracellular concentrations and inhibiting MDR-1 expression in solid tumor cell lines, including the MCF-7 cell line (17), the mechanisms that enable RES to possess a unique antitumor function remain unidentified. The present study hypothesized that reversing the MDR of cancer cells may be an important mechanism.

In the present study, the MCF-7/DOX cell line, characterized by DOX resistance, was used to identify whether RES was capable of reversing the MDR of MCF-7/DOX cells in response to DOX and explore the related mechanism.

## Materials and methods

### Cell culture

MCF-7 and MCF-7/DOX cells (Nanjing KGI Biological Technology Development Co. Ltd., Nanjing, China) were cultured in RPMI-1640 medium (Gibco-BRL, Rockville, MD, USA), which was supplemented with 10% fetal calf serum (Gibco-BRL) at 37°C in a humidified 5% CO_2_ atmosphere. The MCF-7/DOX cells were maintained in a culture medium with or without supplementation of 1.0 μg/ml DOX (Haizheng Medicine Co. Ltd., Zhengjiang, China) two weeks prior to the planned experiments.

### Reversal index (RI) assay

To determine the MDR of the MCF-7/DOX cells to chemotherapeutic agents, the MCF-7 and MCF-7/DOX cells were seeded on 96-well plates (2×10^4^ cells/well) and incubated with various concentrations of DOX (0, 0.01, 0.1, 1, 10 or 100 μM) dissolved in dimethylsulfoxide (DMSO) at a final concentration of 0.1% DMSO. RPMI-1640 culture medium served as a negative control and RPMI-1640 culture medium supplemented with 0.1% DMSO, served as a vehicle control. The cytotoxicity of DOX was measured via an MTT assay (18). Following 48 h of incubation, 200 μl MTT solution (0.5 mg/ml) was added to each well and incubated for 4 h at 37°C. The supernatants were transferred to new 96-well plates and the absorbance was recorded at a wavelength of 570 nm in microplate reader [Multiskan MK3; Thermo Electric (Shanghai) Technology Instrument Co., Ltd., Shanghai, China].

The half maximal inhibitory concentration (IC_50_) was defined as the concentration of the drug that resulted in 50% inhibition of cell growth and was obtained via regression analysis between the drug concentration and cell inhibition rate. The RI value was calculated by dividing the IC_50_ value of the MDR (MCF-7/DOX) cells by the value of the sensitive (MCF-7) cells.

### Intrinsic cytotoxicity assay

The *in vitro* cytotoxicity of RES was measured via an MTT assay. Briefly, MCF-7 and MCF-7/DOX cells at a confluence level of 80–90% were digested and re-seeded on 96-well culture plates (2×10^4^ cells/well). The cells were incubated at 37°C in a 5% CO_2_ atmosphere. Following 24 h, the culture medium was refreshed with RPMI-1640 that was supplemented with various concentrations of RES (4, 8, 12 or 16 μM) dissolved in DMSO with a final concentration of 0.1% (Sigma-Aldrich, St. Louis, MO, USA). RPMI-1640 culture medium served as a negative control and RPMI-1640 culture medium supplemented with 0.1% DMSO, served as vehicle control. As described above, following 48 h of incubation, 200 μl MTT solution (0.5 mg/ml) was added to each well and incubated for 4 h at 37°C. The supernatants were transferred to new 96-well plates and the absorbance was recorded at a wavelength of 570 nm, after which the inhibition ratio (IR) and IC_10_ values were calculated.

### Reversing drug resistance assay

After seeding 1×10^4^ MCF-7/DOX cells per well in a 96-well plate for 24 h, the growth medium was refreshed using a medium that contained RES, DOX or a combination of RES and DOX. Subsequent to 48 h of exposure, the cytotoxicity of the drugs was assessed via an MTT assay. The combinational index (Q) was calculated using the formula: Q=Ea+b/(Ea+Eb-Ea×Eb). Where, Ea+b represented the combinational inhibition rate of RES and DOX and Ea and Eb represented the individual inhibition rate of RES and DOX, respectively. The nature of the drug interaction was defined as: i) Additive (^+^) if Q ranged from 0.85 to 1.15; ii) synergism (^++^) if Q ranged from 1.15 to 2.0; iii) subtraction (^−^) if Q ranged from 0.85 to 0.55; and iv) antagonism (^−−^) when the confidence interval was <0.55. The RI value of RES was calculated by dividing the IC_50_ of DOX by the value of the RES and DOX combination.

### Intracellular accumulation of DOX

MCF-7/DOX cells were cultured in the absence or presence of RES at concentrations of 4, 8, 12 or 16 μM; DOX was added to the cells to obtain a final concentration of 4, 16 or 64 μM. Following 3 h of incubation, the cells were washed three times with ice-cold phosphate-buffered saline (PBS) and incubated in isopropanol overnight at −20°C. The absorbance of the supernatant was read using a fluorescence spectrofluorometer (Hitachi High-Tech Companies, Tokyo, Japan) at wavelengths of 470 and 590 nm. The value of DOX accumulation within the cells was calculated according to the standard curve (19,20).

### Semi-quantitative reverse transcription-polymerase chain reaction (RT-PCR)

Total RNA was extracted from the MCF-7/DOX cells of the different groups (treated with various concentrations of RES, DOX or combinations of RES and DOX) using TRIzol Reagent (Sigma-Aldrich) according to the manufacturer’s instructions. Thereafter, 2 μg total RNA was used to perform first-strand cDNA synthesis (Takara Biotechnology, Co. Ltd., Dalian, China) and PCR was performed using an Applied Biosystems^®^ 7500 RT-PCR analyzer (Carlsbad, CA, USA). The primer sequences were as follows: Forward: 5′-CCCATCATTGCAATAGCAGG-3′ and reverse: 5′-GTTCAAACTTCTGCTCCTGA-3′ for the MDR-1 gene, and the length of the PCR product was 157 bp. The second primer sequence was as follows: Forward: 5′-CACGTCACACTTCATGATGG-3′ and reverse: 5′-ATGTTTGAGACCTTCAACAC-3′ for β-actin, and the length of the PCR product was 496 bp. The amplification conditions were 3 min at 94°C for denaturing, 30 cycles of amplification (94°C for 30 sec, 57°C for 30 sec and 72°C for 1 min) and a cooling step at 4°C. The PCR products were subjected to 1% agarose gel electrophoresis and the spectral density of the bands was visualized and analyzed in a Bandscan 5.0 image analysis system (Glyko Inc., Hayward, CA, USA). The relative gene expression of MDR-1 was determined by normalizing the density of MDR-1 to that of β-actin.

### Western blot analysis

The cells were washed with ice-cold PBS and lysed for 30 min in ice-cold radio-immunoprecipitation assay lysis buffer (20 mM Tris-HCl (pH 7.5), 1 mM EDTA, 1 mM ethylene glycol tetraacetic acid, 150 mM NaCl, 1% Triton X-100 and protease inhibitor cocktail; Sigma-Aldrich, St. Louis, MO, USA). The protein concentration was measured using a bicinchoninic acid protein assay kit (Pierce Biotechnology, Inc., Rockford, IL, USA). The protein samples were separated via SDS-PAGE and electroblotted to polyvinylidene difluoride membranes (Millipore*,* Billerica, MA, USA). The membranes were blocked with Tris-buffered saline (TBS) overnight at 4°C and incubated with primary mouse monoclonal antibodies (Maixin Biotechnology Co. Ltd. Fuzhou, China) against P-gP or β-actin for 2 h at room temperature. Following three washes in TBS with 0.1% Tween-20 (TBST), the membranes were probed with a secondary horseradish peroxidase-conjugated goat anti-mouse antibody (Beijing Zhongshan Golden Bridge Biotechnology Co., Ltd., Beijing, China) for 2 h. Following a further three washes with TBST, the immune complexes were detected by chemiluminescence (KPL Inc., Gaithersburg, MD, USA). The spectral density of the bands was visualized and analyzed using a Bandscan 5.0 image analysis system and the expression of P-gP was obtained by normalizing the density of P-gP to that of β-actin.

### Statistical analysis

Data were expressed as the mean ± standard deviation (n=4). Statistical significance was assessed using one-way analysis of variance with SPSS 19.0 software (SPSS Inc., Chicago, IL, USA) and P<0.05 was considered to indicate a statistically significant difference.

## Results

### RES inhibits the proliferation of MCF-7/DOX and MCF-7 cells

**Figure 1:**
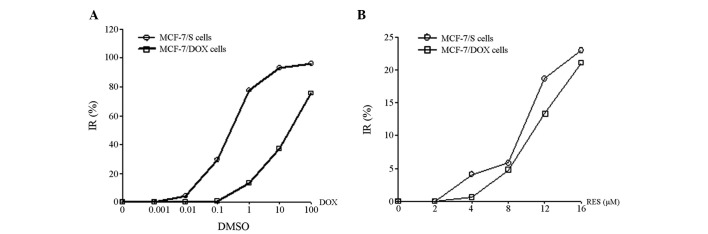
Inhibition of DOX or RES on proliferation of MCF-7/DOX (DOX-resistant cell line) and MCF-7/sensitive (s) cells. MCF-7/DOX and MCF-7/s cells were exposed to serial dilutions of (A) DOX or (B) RES for 48 h and cell inhibition rate was determined via an MTT assay for MCF-7/s (circle) and MCF-7/DOX (square) cells. DOX, doxorubicin; IR, inhibition ratio; DMSO, dimethylsulfoxide; RES, resveratrol.

The IC_50_ values of DOX were 0.39 and 21.38 μM in MCF-7 and MCF-7/DOX cells, respectively (Fig. 1A). The MCF-7/DOX cells were 54.82 times more resistant to DOX, when compared with the MCF-7 cells. RES was identified to be capable of inhibiting the proliferation of MCF-7/DOX and MCF-7 cells; however, no significant difference was demonstrated between the IC_10_ of RES on MCF-7 cells (8.46 μM) and that of MCF-7/DOX cells (11.39 μM; P>0.05). In addition, the proliferation of MCF-7/DOX cells was inhibited by RES in a dose-dependent manner (Fig. 1B).

### RES enhances the cytotoxicity of DOX on MCF-7/DOX cells

**Figure 2:**
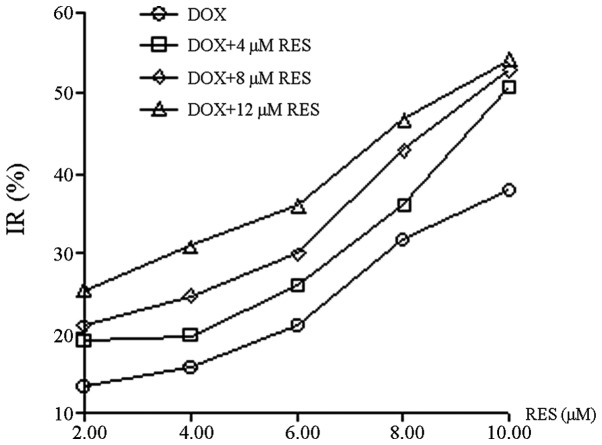
Combinational effect of RES and DOX on inhibiting the cell proliferation of MCF-7/DOX cells (DOX-resistant cell line). The cells were exposed to serial dilutions of DOX or a combination of RES and DOX for 48 h. The cell IR of DOX (circle) and DOX in combination with 4 μM RES (square), 8 μM RES (diamond) and 12 μM RES (triangle) was determined by an MTT assay on the MCF-7/DOX cells. DOX, doxorubicin; RES, resveratrol; IR, inhibition ratio.

RES was demonstrated to inhibit the growth of MCF-7/DOX cells in a dose-dependent manner (Fig. 2); with the increase in RES concentration, the growth of MCF-7/DOX cells gradually decreased. RES at 12 μM exhibited a comparable inhibitory rate (~10%) on MCF-7 and MCF-7/DOX cells, therefore, the concentration of 12 μM was considered to be a non-cytotoxic dose. The reversal effect of RES at a concentration of 12 μM on the MDR of MCF-7/DOX cells was investigated. Q and RI were calculated based on the IR and 50% IC_50_; the values were subsequently used to assess the combinational inhibitory effect of RES and DOX on the MCF-7/DOX cells. RES and DOX were identified to synergistically inhibit MCF-7/DOX cell growth and Q was often >1.15. Moreover, RES enhanced the inhibitory effect of DOX on cell growth in a dose-dependent manner and the RI of DOX was increased from 1.950 to 2.355, as the concentration of RES increased from 4 to 12 μM. The effect of RES on the enhancement of DOX cytotoxicity within MCF-7/DOX cells is shown in Table I.

### RES increases DOX accumulation within MCF-7/DOX cells

**Figure 3:**
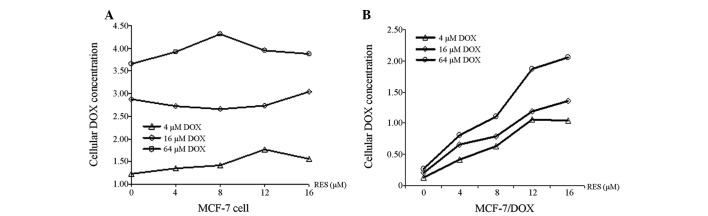
Synergistic effect of RES and DOX on DOX cellular accumulation in MCF-7 and MCF-7/DOX (DOX-resistant MCF-7 cells) cells. (A) MCF-7 cells or (B) MCF-7/DOX cells were exposed to serial dilutions of RES in combination with 4 μM DOX (triangle), 16 μM DOX (diamond) or 64 μM DOX (circle) for 3 h. The cellular DOX concentration was determined by fluorescence spectroscopy. P<0.01, compared with the MCF-7 cells. DOX, doxorubicin; RES, resveratrol.

The capability of RES to promote DOX accumulation within MCF-7 and MCF-7/DOX cells is shown in Fig. 3. The concentration of DOX in MCF-7 (Fig. 3A) and MCF-7/DOX cells (Fig. 3B) increased with increasing DOX treatment, regardless of the RES dose, however, the concentration of DOX in MCF-7/DOX cells was significantly lower than that observed in the MCF-7 cells (P<0.01). In addition, RES was demonstrated to be capable of elevating the concentration of DOX in MCF-7/DOX cells in a dose-dependent manner, however, this did not occur in the MCF-7 cells.

### RES decreases MDR-1 gene and protein expression levels within MCF-7/DOX cells

**Figure 4:**
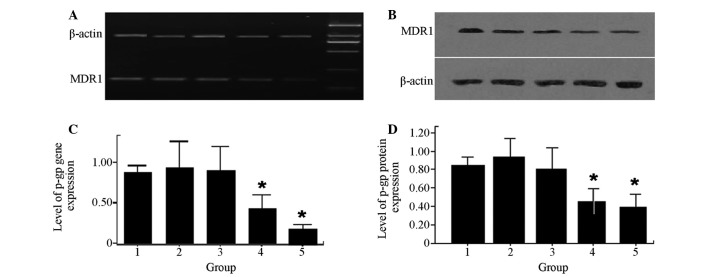
Effects of RES and DOX on MDR-1 gene and protein expression in MCF-7/DOX cells. (A and C) Lane 1, MDR-1 gene expression levels in MCF-7/DOX cells; lanes 2, 3, 4 and 5, treatment with 8 μM DOX, 10 μM RES, 6 μM DOX combined with 8 μM RES, 10 μM DOX combined with 12 μM RES for 48 h, respectively; β-actin, control; lane M, DNA ladder. (B and D) Lane 1, protein expression of P-gP in MCF-7/DOX cells; lanes 2, 3, 4 and 5, treatment with 8 μM DOX, 10 μM RES, 6 μM DOX combined with 8 μM RES, 10 μM DOX combined with 12 μM RES for 48 h, respectively. ^*^P<0.05 compared with lane 1. MDR, multidrug resistance; RES, resveratrol; DOX, doxorubicin.

To determine the mechanism by which RES functionally elevates drug accumulation within MCF-7/DOX cells, MDR-1 mRNA expression was quantitatively measured using RT-PCR. In addition, P-gP, a protein encoded by MDR-1, was quantitatively measured using western blot analysis. The levels of MDRl gene expression (Fig. 4A) and P-gP expression (Fig. 4B) in MCF-7/DOX cells significantly decreased when the cells were treated with a combination of RES and DOX (P<0.05).

## Discussion

MDR is a prevalent issue in cancer chemotherapy, thus, reversing MDR in cancer cells may provide a basis for overcoming drug resistance, and improving chemotherapy and the outcome for cancer patients. RES is hypothesized to possess unique health benefits, including prolonging life, providing cardiovascular protection and exhibiting anti-inflammatory effects (21).

In the present study, the inhibitory effect of RES on human breast cancer cell proliferation was investigated. The results indicated that RES inhibited the proliferation of MCF-7/DOX and MCF-7 cells, which was consistent with previous studies that demonstrated a strong chemopreventive effect of RES against the development of breast cancer (14,15). RES inhibited the growth of human cancer cells *in vitro,* when administered alone or in combination with other anticancer drugs (22,23). Furthermore, the effects of RES on the cytotoxicity of DOX in MCF-7/DOX cells, which exhibited DOX-resistance was investigated in the present study. The RI of MCF-7/DOX cells, relative to DOX and RES treatment, was observed to be significantly higher than that of the group without RES treatment. These results demonstrated that RES enhanced the DOX cytotoxicity effect within MCF-7/DOX cells, which indicated a synergistic effect of RES and DOX.

In addition, the mechanism by which RES enhanced DOX cytotoxicity was investigated. It was identified that, when combined with DOX, RES elevated the concentration of DOX in MCF-7/DOX cells in a dose-dependent manner, while promoting DOX accumulation in the MCF-7/DOX cells. This result provides a partial explanation for why RES may enhance DOX cytotoxicity within MCF-7/DOX cells. The results further revealed that the mRNA and protein expression of the MDR-1 gene were significantly inhibited by RES, indicating that RES enhanced DOX cytotoxicity via downregulating MDR-1 expression. In addition, one of the membrane transport proteins, P-gP was identified in a previous study to promote the expulsion of anticancer drugs, which is considered to be a typical MDR mechanism (24).

In conclusion, the mechanism by which RES exerts its antitumor efficacy remains to be determined. The present study demonstrated that RES inhibited the proliferation of MCF-7/DOX and MCF-7 cells in a dose-dependent manner and significantly enhanced the cytotoxicity of DOX within MCF-7/DOX cells. Moreover, RI was observed to be significantly higher with RES treatment when compared with cells without treatment. In addition, RES reversed MDR in MCF-7/DOX cells, elevated the concentration of DOX within MCF-7/DOX cells and significantly downregulated the expression of the MDR-1 gene and P-gP protein. Therefore, it was concluded that reversing DOX resistance by downregulating MDR-1 expression, is one of the mechanisms that provides RES with a unique antitumor function. Thus, these findings indicate that RES may potentially act as a novel MDR reversal agent for breast cancer therapy.

## Figures and Tables

**Table I:** Antitumor effects of RES combinined with DOX on MCF-7/DOX cells (n=4).

	0 μM RES		4 μM RES			8 μM RES			12 μM RES		
							
	OD	IR (%)	OD	IR (%)	Q	OD	IR (%)	Q	OD	IR (%)	Q
DOX (μM)											
2	0.972±0.094	13.37	0.910±0.012	18.89	1.365	0.887±0.014	20.94	1.196	0.839±0.029	25.22	1.010
4	0.947±0.062	15.60	0.902±0.019	19.61	1.221	0.847±0.034	24.51	1.249	0.775±0.042	30.93	1.149
6	0.888±0.077	20.86	0.831±0.077	25.94	1.219	0.786±0.057	29.95	1.216	0.718±0.040	36.01	1.144
8	0.767±0.088	31.52	0.718±0.075	36.01	1.130	0.640±0.127	42.96	1.235	0.598±0.036	46.70	1.147
10	0.695±0.019	38.06	0.553±0.017	50.71	1.321	0.528±0.039	52.94	1.291	0.513±0.022	54.28	1.171
IC_50_			10.940			9.817			9.077		
RI			1.950			2.178			2.355		

RES, resveratrol; DOX, doxorubicin; MCF-7/DOX, DOX-resistant MCF-7 cells; OD, optical density; IR, inhibition ratio; Q, combinational index; IC_50_, half maximal inhibitory concentration; RI, reversal index.
